# Community structure of thermophilic photosynthetic microbial mats and flocs at Sembawang Hot Spring, Singapore

**DOI:** 10.3389/fmicb.2023.1189468

**Published:** 2023-06-16

**Authors:** Christaline George, Chloe Xue Qi Lim, Yan Tong, Stephen Brian Pointing

**Affiliations:** ^1^Yale-NUS College, National University of Singapore, Singapore, Singapore; ^2^Department of Biological Sciences, National University of Singapore, Singapore, Singapore

**Keywords:** cyanobacteria, Chloroflexia, extremophile, heat stress, hot spring, photosynthesis, polyextremophile, thermophile

## Abstract

The Sembawang Hot Spring in Singapore lies at the foot of a major regional geological feature called the Bentong-Raub Suture Zone. Amid an extensively managed surface geothermal park, an undisturbed hot spring emerges with source water at 61°C, pH 6.8, and 1 mg/L dissolved sulfide. A small main pool at the source supported orange-green benthic flocs, whereas the outflow channel with gradually less extreme environmental stress supported extensive vivid green microbial mats. Microscopy revealed that cyanobacterial morphotypes were distinct in flocs and mats at several intervals along the environmental gradient, and we describe a spiraling pattern in the oscillatorian cyanobacteria that may reflect response to poly-extreme stress. Estimation of diversity using 16S rRNA gene sequencing revealed assemblages that were dominated by phototrophic bacteria. The most abundant taxa in flocs at 61°C/1 mg/L sulfide were *Roseiflexus* sp. and *Thermosynechococcus elongatus*, whilst the mats at 45.7–55.3°C/0–0.5 mg/L sulfide were dominated by *Oscillatoriales* cyanobacterium MTP1 and *Chloroflexus* sp. Occurrence of diverse chemoautotrophs and heterotrophs reflected known thermal ranges for taxa, and of note was the high abundance of thermophilic cellulolytic bacteria that likely reflected the large allochthonous leaf input. A clear shift in ASV-defined putative ecotypes occurred along the environmental stress gradient of the hot spring and overall diversity was inversely correlated to environmental stress. Significant correlations for abiotic variables with observed biotic diversity were identified for temperature, sulfide, and carbonate. A network analysis revealed three putative modules of biotic interactions that also reflected the taxonomic composition at intervals along the environmental gradient. Overall, the data indicated that three distinct microbial communities were supported within a small spatial scale along the poly-extreme environmental gradient. The findings add to the growing inventory of hot spring microbiomes and address an important biogeographic knowledge gap for the region.

## 1. Introduction

Hot springs are created when subsurface tectonic or volcanic activity heats water that rises to the surface. Temperatures range from 40°C to boiling and this restricts the biology of hot springs to a unique group of microorganisms called thermophiles that grow obligately under such conditions (Shu and Huang, 2022). The thermal gradient in hot springs is often accompanied by other stressors such as low/high pH, dissolved sulfide and other minerals, and oligotrophic conditions. Thus, some thermophiles may be considered polyextremophiles adapted to multiple abiotic challenges. At the highest temperatures from approximately 70–80°C to boiling temperatures, specialized communities dominated by Aquificae, Crenarcheaota, and Proteobacteria occur in water and sediments (Shu and Huang, 2022), and as conspicuous streamers (Purcell et al., 2007). At lower temperatures diversity is driven largely by pH for aquatic (Power et al., 2018) and microbial mat communities (Fecteau et al., 2022).

The neutral/alkaline-chloride hot springs have been particularly well studied because they support abundant thermophilic photosynthetic microbial mats at temperatures ≤75°C, and they are usually characterized by their dominant cyanobacterial taxa. From ~60-75°C mats support a thin surface layer of the unicellular cyanobacterial genera *Synechococcus* and/or *Thermosynechococcus*, which have been extensively studied at Yellowstone National Park (YNP) in the United States, e.g., Klatt et al. (2011), Podar et al. (2020), and Kees et al. (2022). Beneath this surface layer much of the mat biomass comprises Chloroflexia and other heterotrophic and autotrophic bacteria and archaea (Martinez et al., 2019). Mats at lower temperatures from ~45 to 60°C are dominated by phylogenetically diverse filamentous cyanobacteria along with other bacteria including Chloroflexia and other archaeal lineages also occur in most mat types: Some mats have a very broad thermal tolerance and are encountered across this thermal range which can be dominated by the genus *Fischerella* (Lacap et al., 2007; Alcorta et al., 2018); and diverse taxa within the Oscillatoriales including sulfide-tolerant ecotypes (Castenholz and Utkilen, 1984; Podar et al., 2020). At lower temperatures of ~45–50°C mats support higher diversity of cyanobacteria including some facultative thermophiles, e.g., *Calothrix*, *Leptolyngbya* and *Nostoc* (Rozanov et al., 2017).

Evidence for endemism and allopatric speciation has been identified across intercontinental distances for hot spring cyanobacteria (Papke et al., 2003), and numerous phylogenetic studies have shown that geographically separated hot spring mats support novel lineages of thermophilic bacteria and archaea, e.g., Jing et al. (2005, 2006) and Lau et al. (2006, 2009). It is therefore reasonable to assume that interrogation of previously undescribed hot springs may yield novel insight that will help to advance the collective knowledge on thermophilic microbial diversity. A regional knowledge gap occurs for southeast Asia where despite a large number of hot springs, few have been described using next generation molecular ecological approaches. Microscopy and DNA fingerprinting studies of hot spring mats in the Philippines and Thailand have described highly diverse *Fischerella*, *Synechococcus* and other cyanobacteria (Jing et al., 2005; Sompong et al., 2005; Lacap et al., 2007). These findings combined with studies in neighboring regions, e.g., China (Tang et al., 2018; Keshari et al., 2022), India (Subudhi et al., 2018), and Japan (Nakagawa and Fukui, 2002); and a recent bioinformatic meta-analysis of hot spring cyanobacterial genomes (Alcorta et al., 2020), suggest that a large unexplored level of taxonomic diversity awaits description for the region.

Sembawang Hot Spring in Singapore has a long history of cultural use and has been described geochemically (Zhao et al., 2001), but its conspicuous photosynthetic microbial mats have yet to be biologically characterized. Exploratory geological drilling and hydrogeological analysis of boreholes up to 100 m in depth indicated a neutral pH and low carbonate groundwater source rising up through granite fault fractures up to 3,000 m in depth to discharge surface water at approximately 70°C (Zhao et al., 2001). Water reaches the surface in a managed park setting where a borehole supplies geothermally heated water for human recreational use. The surrounding parkland supports a single undisturbed spring that erupts in tropical topsoil to form a small pool (the source) which then flows out into a narrow stream (the channel). This creates a clearly defined gradient of aqueous geochemical stressors that comprises temperature and sulfide, and this supports photosynthetic microbial flocs and mats. Here we describe the microbial diversity of these flocs and mats along the poly-extreme environmental gradient, the correlation with abiotic environmental variables, and potential biotic interactions. The findings are discussed in terms of understanding how microbial mats assemble in response to thermal and sulfide stress, and their contribution to a growing global inventory of thermophilic microbial diversity.

## 2. Materials and methods

### 2.1. Field sampling

**Figure 1:**
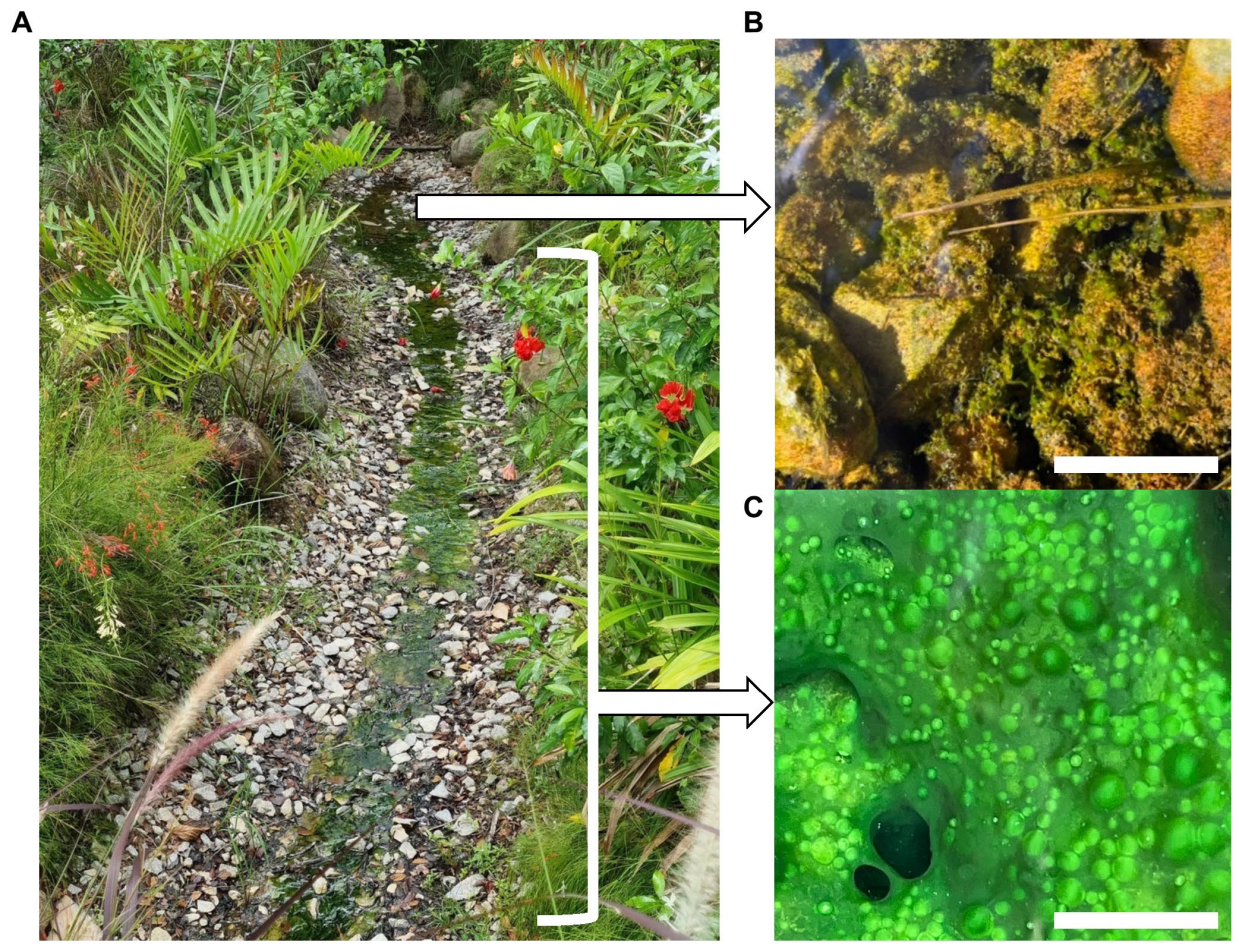
**(A)** Sembawang Hot Spring, **(B)** Floc growth in geothermal source water at 61.4°C, scale bar 20 mm, **(C)** Mat growth in geothermal channel at 45.7–55.3°C, scale bar 10 mm.

The Sembawang Hot Spring lies in a park managed by Singapore National Parks (GPS N 1.434460, E 103.822000). Sampling was conducted at the undisturbed hot spring in March 2022 under National Parks permit NP/RP21-126-1 (Figure 1A). All sampling was conducted using sterilized equipment and persons wore surface-sterilized nitrile gloves and forearm coverings. Biomass was collected from four locations at approximately 5°C increments along the gradient with 10 replicates per location. This included the flocs growing in the geothermal source water at 61.4°C (Figure 1B) and mats growing along the geothermal channel at 45.7–55.3°C (Figure 1C). The collected biomass was placed into screw-cap tubes containing 0.5 mL RNA-later (Thermofisher) nucleic acid preservative solution. Samples were stored on ice in darkness during transport to the laboratory and stored at –20°C until processed within 2 weeks of collection. Abiotic variables relevant to photosynthetic microbial mats were measured in the field as follows: temperature, pH, conductivity (hand-held probes, Hach), alkalinity, carbonate, iron, nitrate, nitrite, phosphate, and sulfide (colorimetric assays, Hach).

### 2.2. Microscopy

The samples were analyzed using confocal microscopy for morphological characterization and through 16S rRNA gene amplicon sequencing for taxonomic diversity estimation. Confocal laser scanning microscopy was performed on a Zeiss LSM900 inverted confocal microscope. Intact and live wet mat and floc samples approximately 1–2 mm^2^ were transferred from the sample tube and sandwiched using glass coverslips. 3D laser scanning confocal was performed with a voxel size of 0.265 μm × 0.265 μm × 1.5 μm by using a Plan-Apochromate 10×/0.45 objective for a 638.90 μm × 638.90 μm sample area at a speed of 0.87 μs/pixel. A 488 nm diode laser was used as an excitation light and signal was collected at 500–700 nm.

### 2.3. DNA sequencing

DNA extraction from biomass samples was carried out using the Qiagen Powerlyzer Powersoil kit with modifications developed by our lab to optimize recovery from photosynthetic microbial mats. The modifications included a manual grinding step prior to extraction using a sterile a pestle and an extended initial lysis step at 70°C for 10 min after the addition of the lysis buffer solution C1, in order to maximize DNA recovery from cyanobacteria that have thick cell walls. The extracted DNA was quantified using the Qubit 1x dsDNA HS Assay Kit (Thermo Fisher), and then processed for amplicon sequencing (Illumina Novaseq PE250, paired end sequencing yielding 2× 250 bp reads). Taxonomic diversity was estimated using 16S rRNA gene amplicon sequencing of the V4 region, using the following primers: 515F (GTGCCAGCMGCCGCGGTAA) and 806R (GGACTACHVGGGTWTCTAAT) (Caporaso et al., 2012). This primer pair was selected because despite the biases that are inherent in any set of primers, these V4 primers have been widely used in microbial ecology and thus they allow broad comparisons to be made (Thompson et al., 2017). The DADA2 (release 1.26) workflow was used to process and analyze the raw sequence reads into amplicon sequence variants (ASVs) (Callahan et al., 2016), and the ASVs were taxonomically annotated using the SILVA 138.1 database (Quast et al., 2013). Any ASVs that remained taxonomically unassigned at class level were grouped as ‘Others.’

### 2.4. Statistical analysis

All statistical analyses were performed in the R environment, version 4.2.2 (R Core Team, 2021). Alpha diversity indices for the ASVs including Chao 1 richness, Shannon’s index (H), the Gini-Simpson’s Index (1-D), and Pielou’s evenness (J) were calculated using the vegan package version 2.6-4 (Oksanen et al., 2020). A one-way ANOVA testing was used to assess if alpha diversity varied significantly across the four locations and a *post hoc* pairwise comparison using the Tukey’s Honest Significant Difference (HSD) was conducted to further assess which locations varied significantly. For further analysis, only those ASVs with relative abundances ≥1% in at least one sample were retained. Patterns of beta diversity for ASVs were robust across multiple taxonomic levels and plots are shown variously at ASV, Class, or Phylum level for visual clarity. The ComplexHeatmap package (release 3.16) (Gu et al., 2016; Gu, 2022) was used to display relative abundances of taxa at the phylum and class levels. The indicspecies package version 1.7.12 (De Cáceres et al., 2010) using the multipatt command with the “r.g” function was used to identify ASVs with a mean relative abundance ≥5% which significantly contributed to differences in taxonomic composition between locations using measures of correlation and abundance. The strength of compositional variations was assessed using permutational Multivariate Analysis of Variance (perMANOVA) on the Bray-Curtis pairwise community dissimilarity distances with the adonis function in vegan. Any significant variations in beta diversity assessments were then represented on a non-metric multidimensional scaling (NMDS) ordination plot. The Envfit function in vegan was used to conduct multiple regressions of abiotic variables against ordination axes for biotic data, in order to infer potential influence of abiotic variables on community composition. The variables having a significant impact (*p*-value ≤ 0.001) were then fitted onto the NMDS plot. A network analysis of microbial associations at the hot spring was conducted and displayed with the NetCoMi package version 1.1.0 (Peschel et al., 2021). The Pearson’s correlation between ASVs was used as an association measure which was normalized using the Centered Log-ratio Transformation (CLR) method to avoid compositional effects inherent with sequencing data. The data was then “signed” transformed into dissimilarities to better emphasize strong positive correlations (i.e., higher edge weights than strong negative correlations). The resultant dissimilarities were used for generation of the microbial network.

## 3. Results and discussion

### 3.1. Site description

Cohesive but unstructured microbial mats occurred attached to rock surfaces in the flowing channel from 45.7–55.3°C, 0–0.5 mg/L sulfide, whilst at the highest temperature (61.4°C) and sulfide concentration (1 mg/L) at the source the biomass comprised poorly cohesive benthic flocs (Figure 1). Carbonate was slightly elevated at the source (80 mg/L) compared to the channel (40 mg/L), but we did not identify this as a major stressor to microbial communities. Other measured variables were consistent along the geothermal gradient: pH (pH 6.8), alkalinity (40 mg/L), phosphate (100 ng/L), with undetectable nitrate, nitrite, or iron levels. The high conductivity (1,900–1,925 μS) along the geothermal feature relative to surrounding terrestrial aquatic habitats has been attributed to seawater mixing with subsurface water (Zhao et al., 2001), although the conductivity was not unusually high compared with other hot springs, e.g., Upin et al. (2023). We therefore identified a poly-extreme gradient comprising temperature (45.7–61.4°C), and sulfide (0–1 mg/L) (Supplementary Table S1).

### 3.2. Morphological characterization of mats and flocs

**Figure 2:**
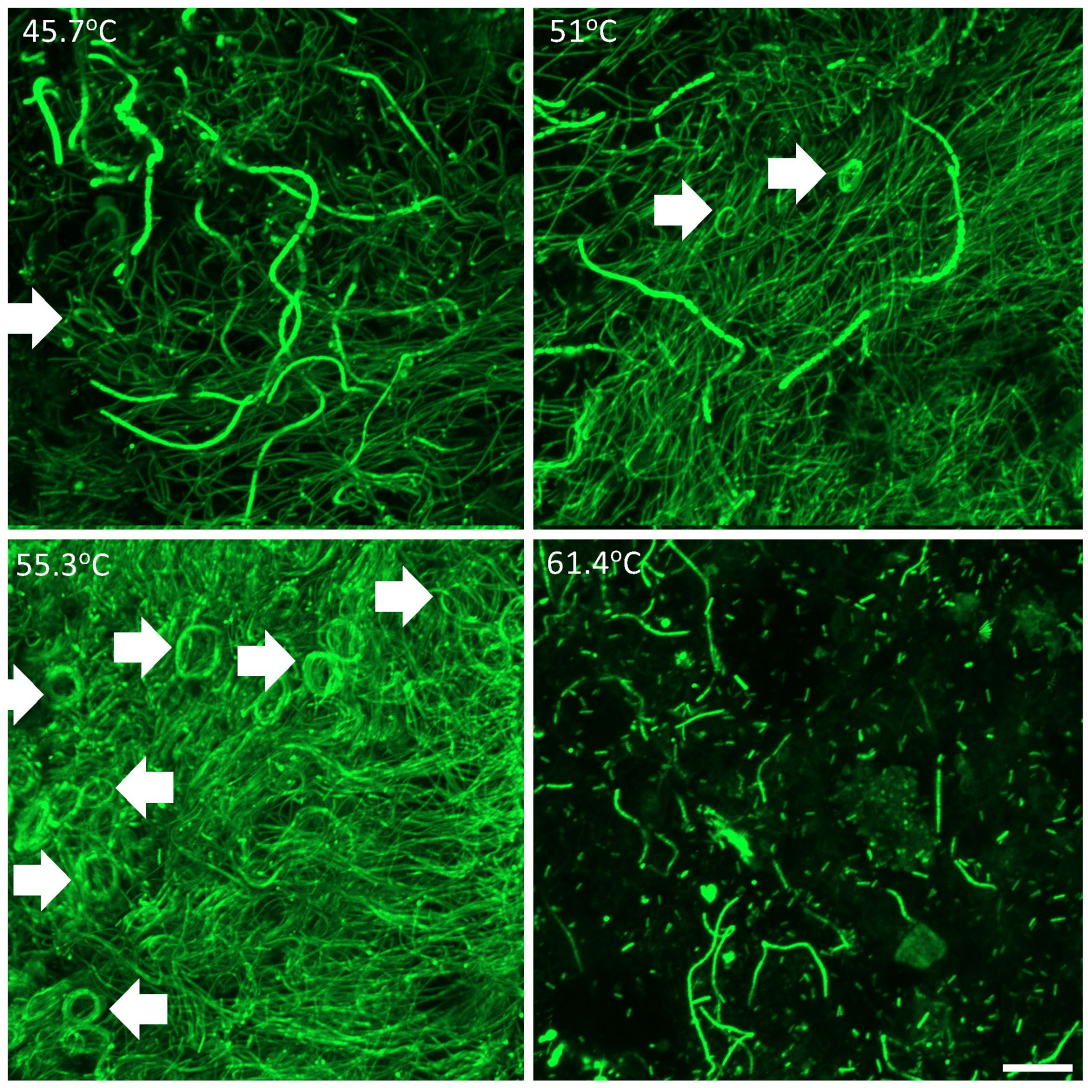
Confocal laser scanning microscopy showing three-dimensional organization of cyanobacterial filamentous and unicellular morphotypes within mats at 45.7–55.3°C and floc at 61.4°C. Arrows denote spiraling structures of oscillatorian morphotypes. Scale bar 20 mm.

Thermophilic photosynthetic microbial mats have traditionally been categorized according to their cyanobacterial composition, we therefore first conducted morphological observation of cyanobacteria using laser confocal microscopy (Figure 2). The lowest temperature (45.7°C) sulfide-free location supported diverse filamentous and unicellular cyanobacterial morphotypes. At 51–55.3°C and 0.3–0.5 mg/L sulfide oscillatorian cyanobacterial morphotypes were dominant. At 61.4°C and 1 mg/L sulfide cyanobacteria were less abundant and dominated by unicellular *Synechococcus*-like morphotypes, although fragmented filamentous cyanobacteria were also present. An interesting observation was the spiral filamentous aggregations of the oscillatorian cyanobacterial morphotypes. This growth pattern (also known as doughnuts) has been observed *in vitro* for other oscillatorian cyanobacteria resulting from restricted movement and reflective of its interaction with the immediate environment (Sato et al., 2014). A study on spiraling in the aquatic cyanobacterium *Arthrospira platensis* revealed that thermal and UV stress increased spiraling and this morphological change together with increased production of an unidentified 52 kDa periplasmic protein resulted in increased tolerance to thermal and UV stress along with enhanced photochemical yield (Ma and Gao, 2009). We therefore hypothesize that this feature may also reflect a response to the poly-extreme stress gradient in Sembawang Hot Spring, with a qualitative estimation that spiraling was observed more frequently in samples under elevated stress, i.e., at higher temperature and sulfide, and attachment to a rock substrate which may impose restrict movement of the filaments (Figure 2). This study did not conduct microscopy of Chloroflexia that are common components of hot spring microbial mats and are also known to be motile. Since mats are usually dominated by these two phototrophic bacterial groups, it is possible that motility may play an important role in facilitating mats to physically respond to stress exposure along micro-habitat gradients in hot springs.

### 3.3. Estimation of taxonomic diversity in mats and flocs

**Figure 3:**
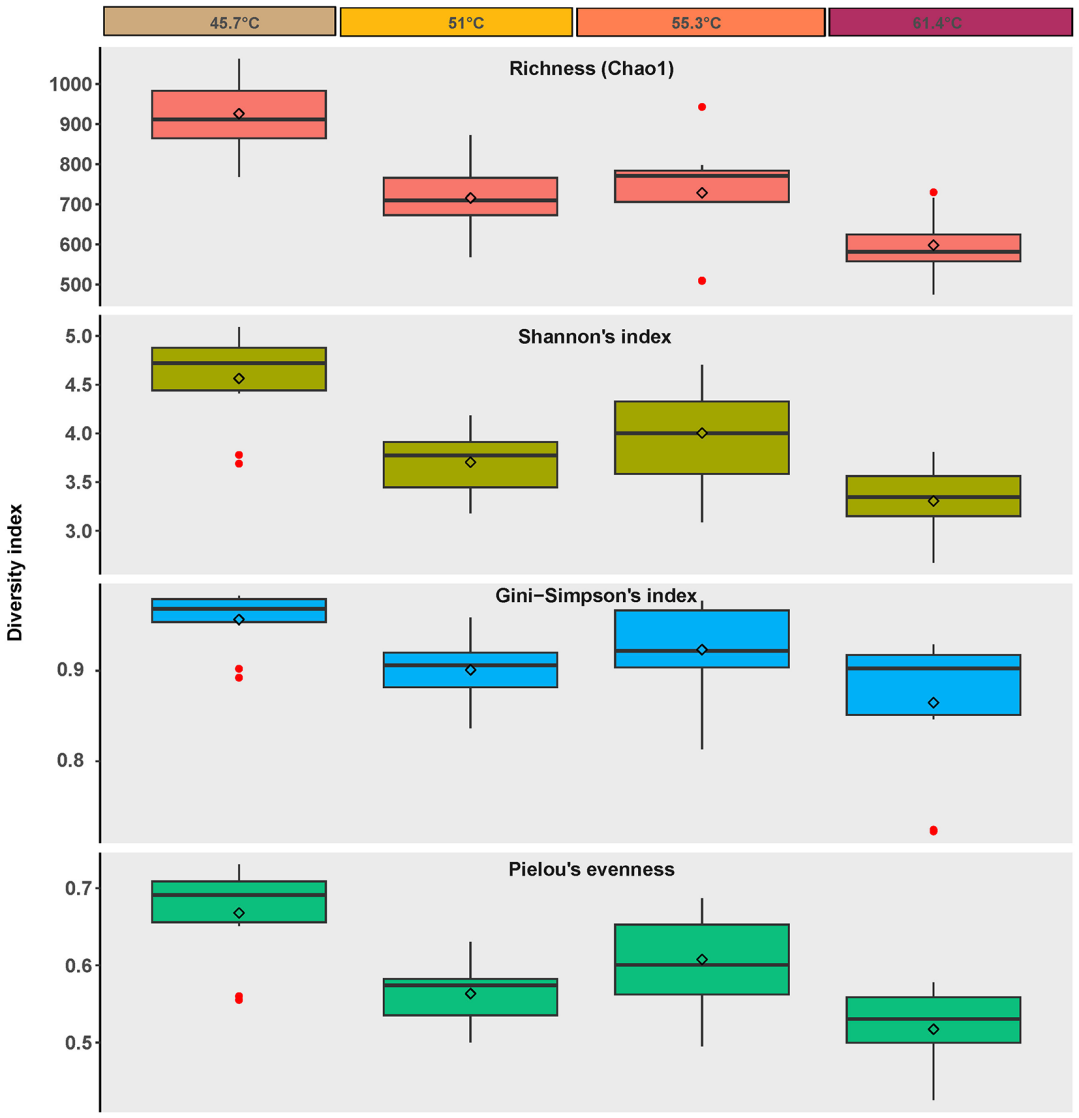
Alpha diversity metrics for ASVs recovered from photosynthetic microbial mats at 45.7–55.3°C and flocs at 61.4°C, diamonds = mean, bar = median, box = interquartile range, error bars = minimum and maximum values. Red dots indicate values outside the interquartile range.

Diversity estimation using high throughput sequencing of the V4 region of the 16S rRNA gene revealed a highly diverse community (3,540 ASVs overall, with 1,063 ASVs in the most diverse sample A4 at 45.7°C, and 475 AVSs in the least diverse sample D9 at 61.4°C) across the environmental gradient of the hot spring (Figure 3). A significant inverse correlation with the gradient of temperature and sulfide stress was observed for: Species richness (using Chao 1 to estimate species number) (one-way ANOVA *p* = <0.001, *post hoc* Tukey *p* = <0.05), Shannon’s Index (i.e., diversity estimated with emphasis on rare taxa) (one-way ANOVA *p* = <0.001, *post hoc* Tukey *p* = <0.05), Gini-Simpson Index (i.e., diversity estimated with emphasis on common taxa) (one-way ANOVA *p* = 0.004, *post hoc* Tukey *p* = <0.01), and Pielou’s Eveness (i.e., how equally the taxa are represented) (one-way ANOVA *p* = <0.001, *post hoc* Tukey *p* = <0.01). Thus, indicating a trend toward a less diverse and more specialized community with increasingly poly-extreme stress (i.e., decreased richness with increased dominance). The decrease in species richness with increasing thermal stress has been observed for other geothermal springs and our observations are consistent with this well-established phenomenon, e.g., Nakagawa and Fukui (2002) and Miller et al. (2009), although we also note that for some hot spring systems the relationship between diversity and thermal stress was not linear (Lau et al., 2006; Podar et al., 2020).

**Figure 4:**
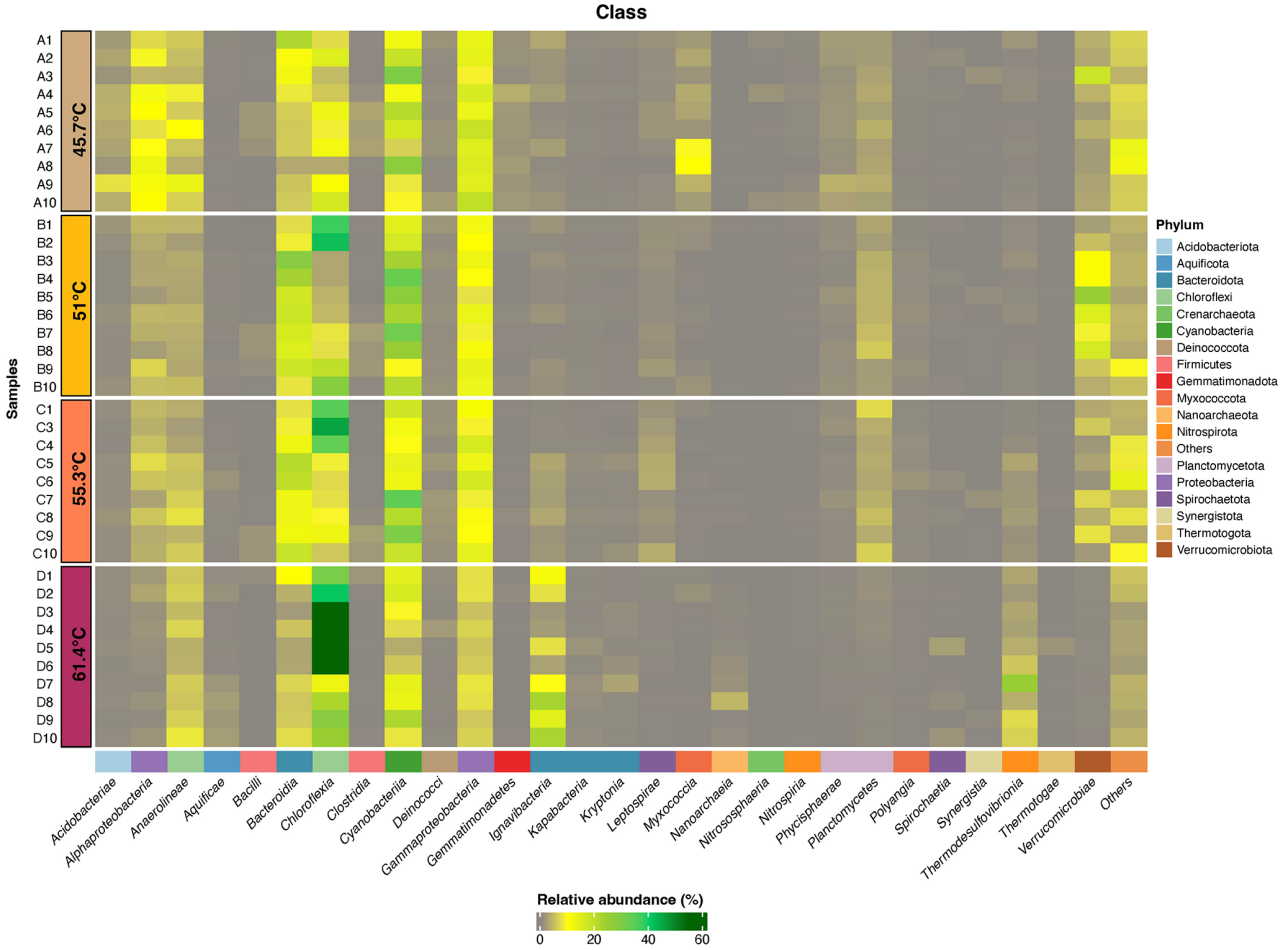
Heatmap displaying distribution of ASVs (≥1% relative abundance) at Phylum and Class levels for photosynthetic microbial mats at 45.7–55.3°C and flocs at 61.4°C. ASVs that were assigned to a Phylum but could not be definitely assigned to a Class are shown as Others. Taxonomic identity of all ASVs is shown in Supplementary material.

**Figure 5:**
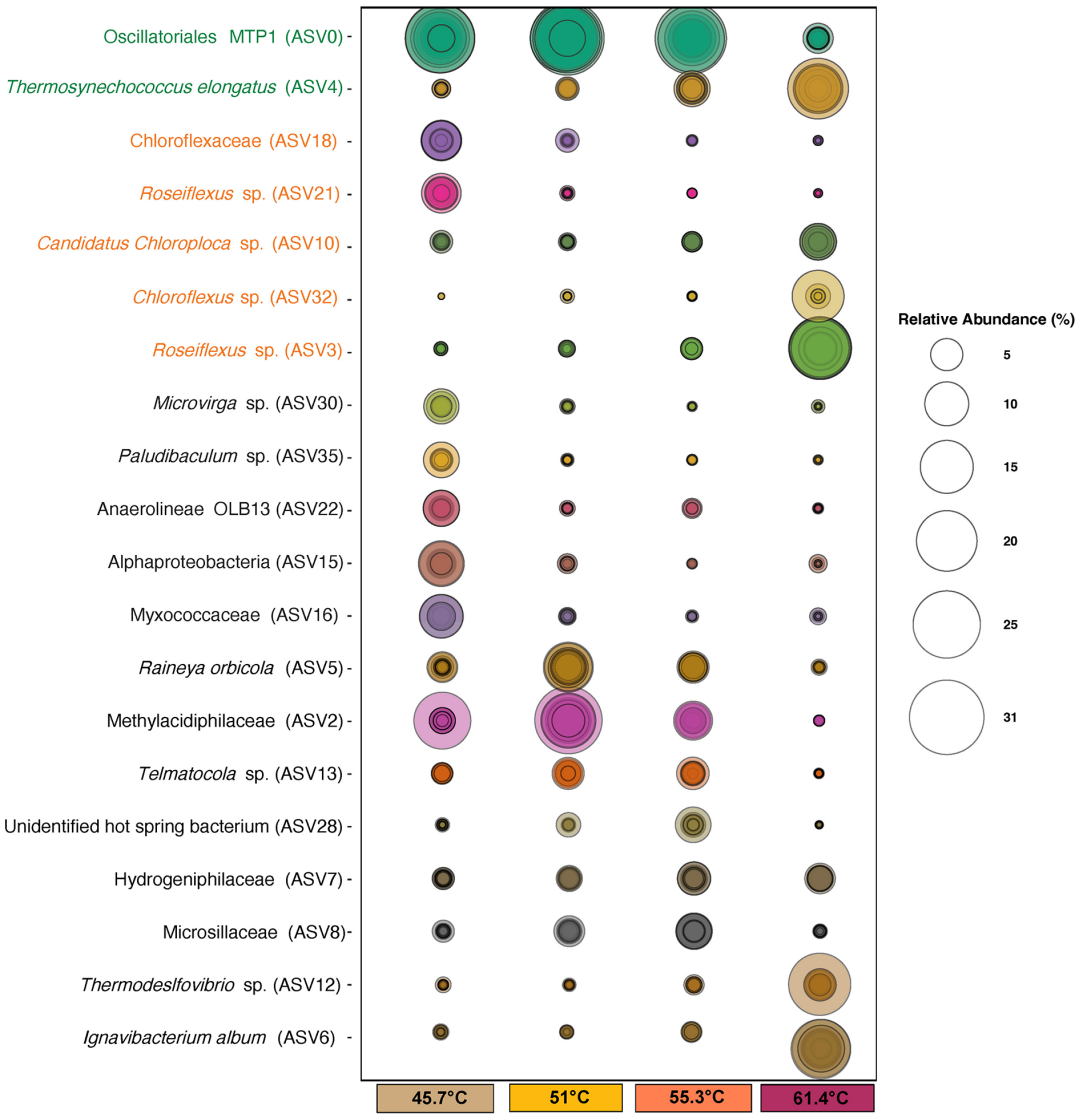
Bubble plot of identified ASVs at ≥5% relative abundance that were significant (*p* = <0.05) in explaining differences in occurrence/abundance between mats at 45.7–55.3°C and flocs at 61.4°C. Size of circles represents the relative abundance of taxa. Concentric circles indicate values for individual samples. ASV names in green font denote Cyanobacteria, and those in orange font denote Chloroflexia. ASV identities were assigned using the SILVA 138.1 database.

To gain further insight on the dominant taxa in the community we focused subsequent analysis on ASVs comprising ≥1% relative abundance. These resolved taxonomically into 18 phyla, comprising 27 bacterial classes and one archaeal class (Figure 4, Supplementary Table S2, and Supplementary Figure S1). We acknowledge that primer bias may have affected our estimations however this is an inherent problem with any PCR-based interrogation. Overall, the Cyanobacteria were most abundant in mats between 45.7 and 55.3°C, whilst in the flocs at 61.4°C the Chloroflexia were most abundant. Among the classes that support heterotrophic and chemoautotrophic taxa the Bacteroidia and Gammaproteobacteria were most abundant in mats whilst in flocs the Ignavibacteria were most abundant. Other classes displayed less abundant representation. In order to identify the ASVs that were most influential in explaining the different assemblages between sample locations we conducted multiple correlation analysis (Indicspecies) while excluding ASVs with relative abundance <5% in at least one sample, to identify taxa that were significantly different in occurrence/abundance between locations (*p* = <0.05; Figure 5). The patterns in occurrence are described in detail below.

The most abundant ASVs belonged to photosynthetic bacteria within the Cyanobacteria and Chloroflexia. Amongst the cyanobacteria the filamentous novel Oscillatoriales cyanobacterium MTP1 (ASV0) (Hallenbeck et al., 2016) was most abundant in mats, whereas in the flocs at highest stress levels it was largely replaced by unicellular *Thermosynechococcus elongatus* (ASV4) as the dominant oxygenic photoautotroph and this was consistent with our microscopy observations. Other cyanobacteria (ASV45, *Geitlerinema,* ASV87 and ASV 98 *Leptolyngya*, and ASV24 *Fischerella*) occurred at low abundance and their distribution reflected a preference for lower temperatures. For the Chloroflexia, distinct low and high temperature/sulfide ecotypes for both *Chloroflexus* (ASV18 and ASV32) and *Roseiflexus* (ASV3 and ASV21) were suggested from the abundance patterns of the ASVs. At the highest temperature another Chloroflexia ASV, *Candidatus Chloroploca* (ASV10) was also elevated in abundance. Other photosynthetic bacterial groups such as the Chlorobi had low relative abundance (≤1%).

The abundance of phototrophic bacteria suggested that the major source of primary production in this system is via bacterial photoautotrophy, and this has been shown to be achieved by both cyanobacteria and Chloroflexi in hot spring microcosms using isotope incorporation experiments (Schuler et al., 2017; Bennett et al., 2020). Some Cyanobacteria are also capable of dinitrogen assimilation and so they may be important routes for nitrogen input to the system. Some well-known nitrogen-fixing hot spring cyanobacteria, i.e., *Fischerella*, have been shown to fulfill the nitrogen requirements of mat communities (Estrella Alcaman et al., 2015), however at the Sembawang Hot Spring they occurred at very low abundances (≤1%) at the time of sampling. Although we noted that qualitative microscopy observations from sampling at other times of year suggest *Fischerella* may at times become temporarily more abundant in Sembawang mats, as observed for free-floating cyanobacterial mats in tropical locations subject to seasonal monsoon rains (Lacap et al., 2007). Some *Synechococcus* ecotypes have also been demonstrated to carry out nitrogen fixation under specific environmental conditions, e.g., temperatures above 60°C, anoxic conditions, and low light (Steunou et al., 2006), and so we speculate that some of the ecotypes we encountered in this study may be capable of nitrogen fixation although this requires further testing and evaluation.

Among the non-phototrophic groups, some weakly thermophilic/thermotolerant ASVSs were important in defining the difference in community between 45.7°C and higher temperatures, including *Anaerolineae* OLB13 (ASV22), *Microvirga* sp. (ASV30), *Myxococcaceae* taxon (ASV16), *Paludibaculum* sp. (ASV35), and an unidentified Alphaproteobacteria taxon (ASV15). The OLB13 and *Paludibaculum* taxa suggest fermentative heterotrophy may be important at this lowest temperature, whilst *Microvirga* is an aerobic chemoheterotroph with weakly thermophilic ecotypes (Li et al., 2020). The abundant ASVs encountered at 45.7°C are also common taxa in soil microbiomes (Thompson et al., 2017), and so they may also indicate recruitment from mesophilic surroundings. At mid-range temperature/sulfide levels the non-phototrophic taxa were dominated by thermophilic chemolithoautotrophic taxa including *Methylacidiphillaceae* and *Hydrogenophillaceae* taxa (ASV2, ASV7), as well as aerobic chemoorganotrophs including the widely distributed *Rayneia orbicola* (ASV5) (Albuquerque et al., 2018), and the cellulolytic taxon *Telmatocola* sp. (ASV13) (Rakitin et al., 2021).

The most abundant non-phototrophic taxa at the highest temperature/sulfide level were *Ignavibacterium album* (ASV6), *Thermodesulfovibriona* sp. (ASV12), and a Hydrogenophillaceae bacterium (ASV7) (Figure 5). *Ignavibacterium album* is known for thermophilic anaerobic cellulose degradation (Lino et al., 2010), and so this and the occurrence of other putatively cellulolytic ASVs at lower temperatures, e.g., *Fervidobacterium riparium* (ASV155), *Telmatocola* sp. (ASV13) suggested cellulose may be an important substrate in hot springs such as Sembawang where we observed large allochthonous input of leaves and wood from the surrounding tropical forest. In the flocs it may also in part reflect their co-occurrence with *Thermosynechococcus* since related taxa have been shown to produce cellulose as a component of their glycocalyx and cell wall (Podosokorskaya et al., 2011; Zhao et al., 2015). The abundant *Thermosdesulfovibriona* and hydrogen *Hydrogenophillaceae* ASVs reflected the importance of chemolithoautotrophic pathways to the community at the highest temperature/sulfide levels. Also of note was the recovery of Nanoarchaeaota (*Woesarchaeales* ASV68) in flocs at the highest temperature/sulfide levels. This group possesses greatly reduced genomes and cell sizes, large membrane-associated proteins implicated in attachment to other prokaryotic cells and occur attached to larger archaeal cells and so they are likely to represent taxa with parasitic lifestyles (Wurch et al., 2016).

### 3.4. Beta diversity patterns in mats and flocs

**Figure 6:**
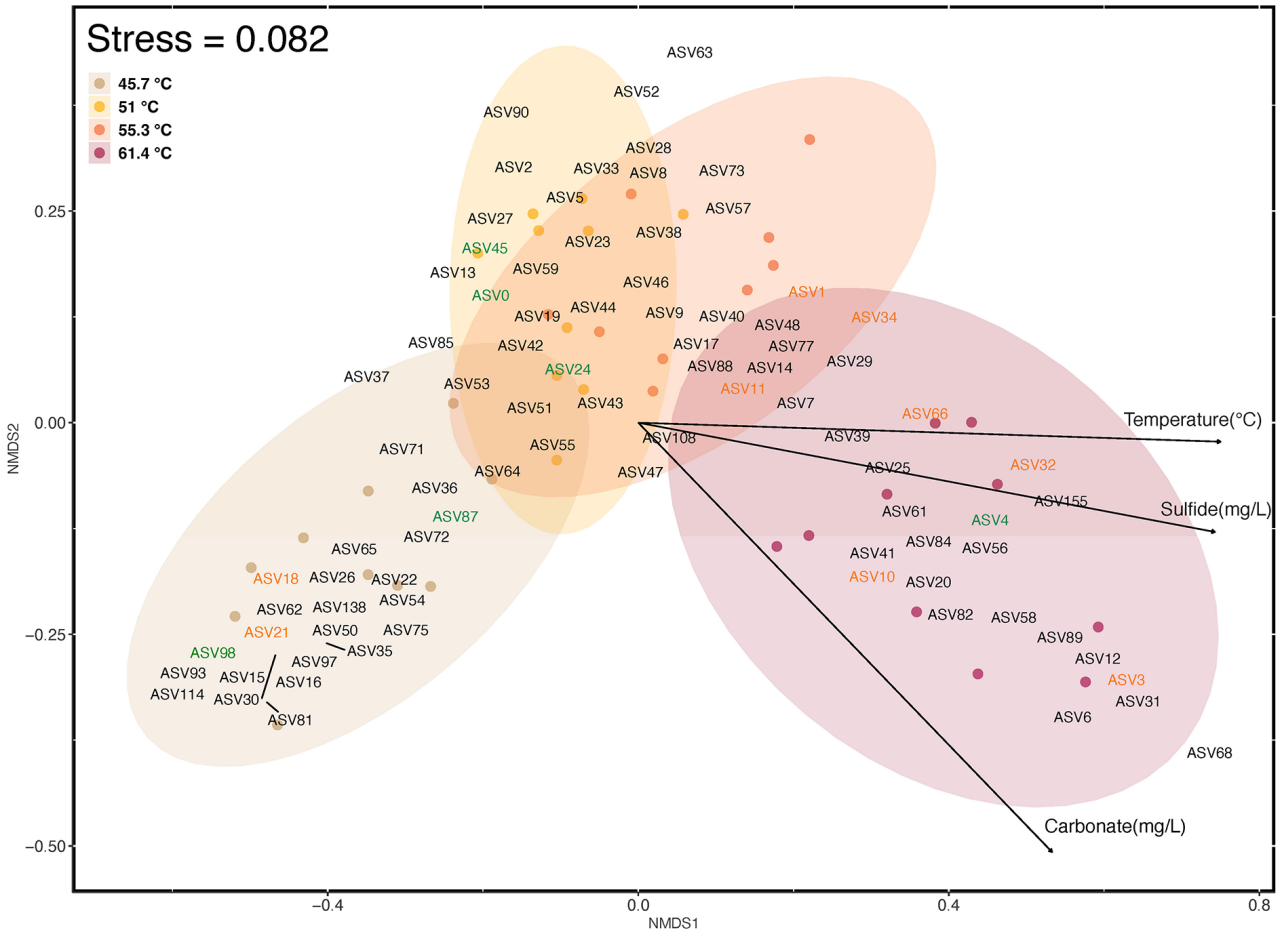
Beta diversity of ASVs (≥1% relative abundance) in photosynthetic microbial mats at 45.7–55.3°C and flocs at 61.4°C, and correlation with abiotic variables as visualized using NMDS of Bray Curtis distances. Colored elipses indicate the four locations along the geothermal gradient, and small colored circles represent ordination placement for each assemblage sampled. ASVs in green font represent Cyanobacteria (ASV0, Oscillatoriales MTP1; ASV4, *Thermosynechococcus elongatus*; ASV24; *Fischerella* PCC9339; ASV45, *Geitlerinema* PCC8501; ASV87, *Leptolyngya* ANT-LT52.2; LT ASV98, *Leptolyngbya* sp.), and those in orange font represent Chloroflexia (ASV1, *Chloroflexus* sp.; ASV3, *Roseiflexus* sp.; ASV10, *Candidatus Chloroploca* sp.; ASV11, *Chloroflexus auriantacus*; ASV18, Chloroflexaceae unidentified sp.; ASV21, *Roseiflexus* sp.; ASV32, *Chloroflexus* sp.; ASV34, *Chloroflexus* sp.; ASV66, *Roseiflexus* sp.). The influence of abiotic variables (*p* ≤ 0.001) was estimated using Envfit analysis and respresented as vectors with arrows on the ordination plot. The direction and length of the arrows indicates direction and maximum magnitude of correlation of the samples to the respective variable. Taxonomic identity and distribution of all ASVs is shown in Supplementary material.

Visualization of beta diversity using Bray Curtis dissimilarities using NMDS ordination indicated the differences in assemblage composition along the gradient (Figure 6). A significant difference occurred between mats in the lower channel with least environmental stress and the flocs at the source with highest poly-extreme stress (one-way permANOVA, *p* = <0.001), but a lack of significant difference between mats in intermediate conditions. This indicated that three potential communities occupied the hot spring environmental gradient. The ordinations also highlighted the value of cyanobacterial ASVs in delineating mat types at lower temperature with 45.7°C supporting *Leptolyngya* in mats, and at 51–55.3°C comprising Oscillatoriales MTP1 mats. This was followed by a shift toward Chloroflexia ASV diversity as a more relevant indicator of community shift at higher temperatures from 55.3 to 61.4°C with *Thermosynechococcus elongatus* as the sole cyanobacterium at the highest temperature.

The correlation of abiotic variables with beta diversity indicated that temperature, sulfide, and carbonate concentrations were significant factors (*p* = <0.001; Figure 6). Temperature is a well-established stressor in hot springs from many locations, e.g., Nakagawa and Fukui (2002), Lau et al. (2006), Miller et al. (2009), and Podar et al. (2020). The sulfide levels encountered in our study may be considered moderate to high for circum-neutral hot springs with values up to 1 mg/L (approximately 29.3 μM), when compared to a survey of over 400 YNP hot springs (Boyd et al., 2012). Some hot spring cyanobacteria display sensitivity to sulfide at levels as low as 0.15 mg/L (approximately 4.4 μM) (Castenholz, 1976), although others and particularly oscillatorian cyanobacteria are notably sulfide-tolerant (Castenholz and Utkilen, 1984; Martin-Clemente et al., 2022). Among the Chloroflexi some taxa may tolerate up to millimolar levels (Kawai et al., 2019). In a study using *in situ* microcosms at YNP acidic and neutral hot springs, for the neutral hot spring (68.6°C, pH 7.12, S_2_ < 150 nM) addition of sulfide to microcosms at a final concentration of 5 μM had no impact on light-driven carbon fixation in mats dominated by *Synechococcus* and *Roseiflexus* (Boyd et al., 2012). Interestingly, the reduction in dissolved sulfide in hot springs with distance from source has been shown to decrease by more than can be explained through abiotic processes alone, and so biological sulfide oxidation may also be important in defining such gradients (Cox et al., 2011).

**Figure 7:**
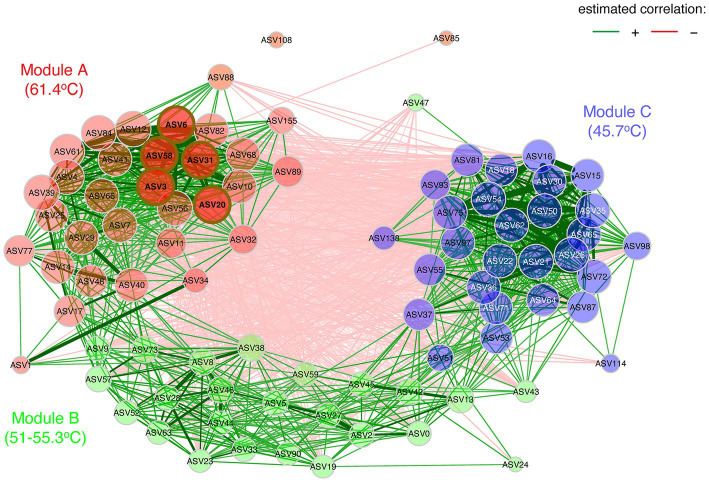
Co-occurrence network analysis based upon Pearson correlations as an association measure for ASV occurrence (≥1% relative abundance), for taxa encountered in mats at 45.7–55.3°C and flocs at 61.4°C. Green lines indicate positive correlations, red lines indicate negative correlations, and line thickness (edge weights) indicate strength of correlation. Eigenvector centrality was used for defining hubs (nodes with a centrality value above the empirical 95% quantile). Node colors represent clustered modules, determined using greedy modularity optimization. Hubs are highlighted by brown borders. Hub ASVs for Module A comprised *Roseiflexus* (ASV3), a denitrifying Anaerolineaceae taxon (ASV20), two *Ignaviabcterium albans* (ASV6, ASV31), and *Thermodseulfovibrio* (ASV58). Module B included Oscillatoriales MTP1 (ASV0), *Geitlerinema* (ASV45), and associated taxa. Module C included two *Leptolyngya* taxa (ASV87, ASV98), and associated taxa. Taxonomic identity and distribution of all ASVs is shown in Supplementary material.

In addition to abiotic influence on community structure, biotic interactions between taxa can also be important determinants. We therefore conducted a network analysis of Pearson correlations for taxa co-occurrence to visualize potential relationships (Figure 7). This showed three distinct modules with strong positive correlations of association between taxa, with module A clustered around ASVs occurring at the most extreme conditions in the source pool at 61.4°C and 1.0 mg/L sulfide, whilst module B comprised AVSs from 51–55.3°C and 0.3–0.5 mg/L sulfide, and module C generally clustered around ASVs from the lowest temperature (45.7°C) sulfide-free location. The distinct associations within modules and relatively few inter-module interactions further reinforced that they represented three distinct communities along the poly-extreme gradient at Sembawang Hot Spring. The network analysis identified five hub ASVs for the high-temperature module, comprising *Roseiflexus* (ASV3), a denitrifying Anaerolineaceae taxon (ASV20), two *Ignaviabcterium albans* (ASV6, ASV31), and *Thermodseulfovibrio* (ASV58). This indicated that anoxygenic phototrophy and anaerobic metabolism may be important in benthic flocs. The association of cyanobacterial AVSs in the two lower temperature modules, namely Oscillatoriales cyanobacterium MTP1 (ASV0) and *Geitlerinema* (ASV45) in module B, and two *Leptolyngya* (ASV87, ASV98) in module C were congruent with the ordinations of community dissimilarity and further supported the delineation of thermophilic mats by their cyanobacterial taxa. The *Fischerella* ASV (ASV 24) displayed few connections and this may reflect its low relative abundance in mats at the time of sampling.

### 3.5. Comparison of different mat layers

**Figure 8:**
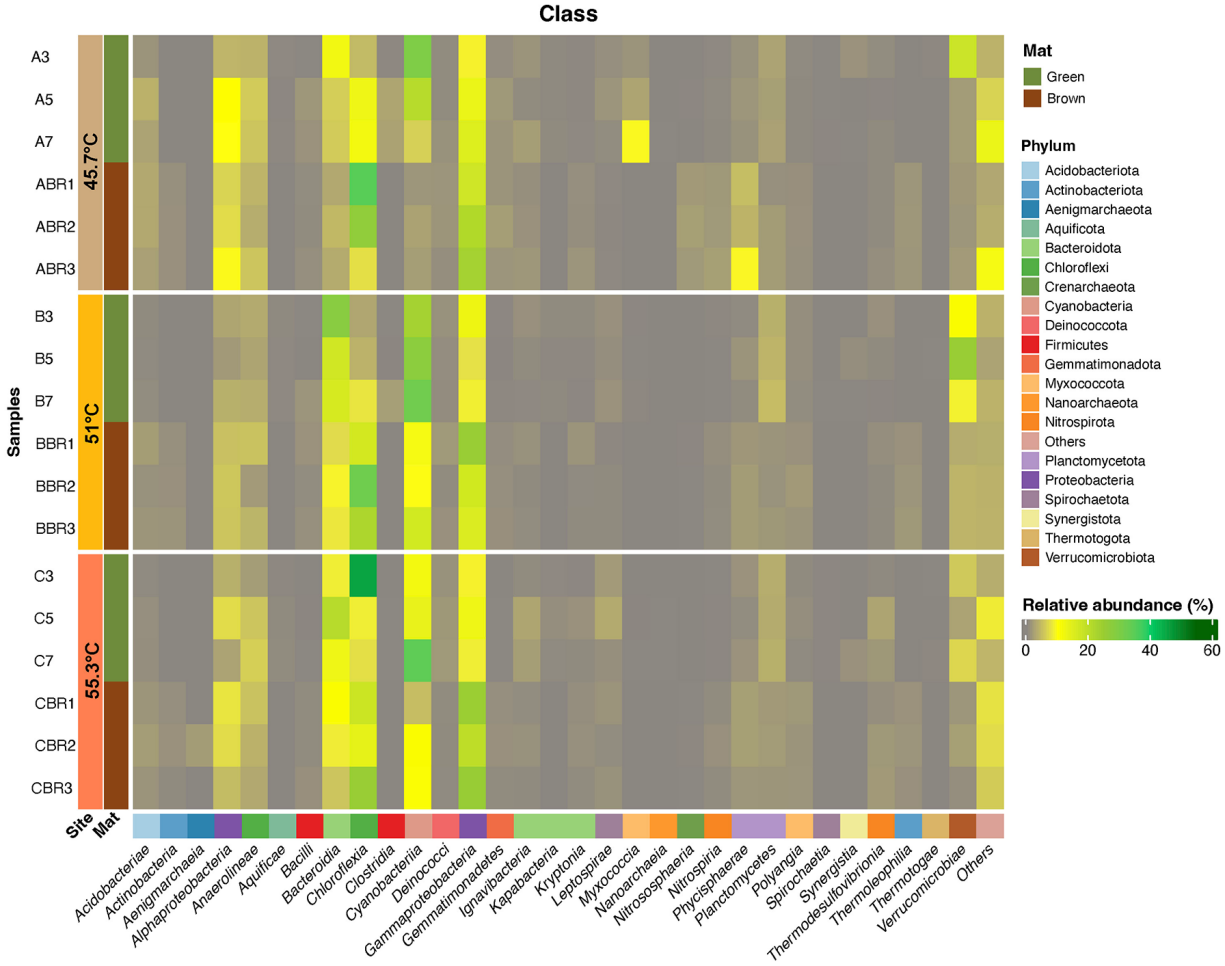
Heatmap displaying distribution of ASVs (≥1% relative abundance) at Phylum and Class levels from green and brown layers of photosynthetic microbial mats occurring at 45.7–55.3°C. ASVs that could not be assigned to a Class are shown as Others. Taxonomic identity of all ASVs is shown in Supplementary material.

A further observation was made that some but not all mats had a distinct brown layer beneath the green mat that was in direct contact with the rock substrate. This was not observed for flocs on submerged rocks. We hypothesize from visual observations that the brown mat layers were micro-oxic or anoxic but this was not measured. We compared the diversity in the brown layers to that of the corresponding green mat directly above (Figure 8). This revealed that brown mats had lower abundance of taxa belonging to the Cyanobacteria and Bacteroidia, and elevated relative abundance of Chloroflexia and Gammaproteobacteria. Similar layers observed in mats from hot springs at YNP were also dominated by *Roseiflexus* (Thiel et al., 2016). The Chloroflexia reflected those present in the upper green mat layer, but this group is known to display considerable physiological plasticity spanning photoautotrophy, photoheterotrophy, and heterotrophy (Hanada, 2014), hence they may perform different roles in the green and brown mat layers. The Gammaproteobacteria included several *Thiobacillus* ASVs that are autotrophs capable of oxidizing hydrogen sulfide present in the hot spring water (Wang et al., 2018).

### 3.6. Adaptation of phototrophic bacteria to hot springs

The limits of thermophily in circum-neutral pH hot springs for the Cyanobacteria and Chloroflexota are well-established from numerous studies in various regions, e.g., Nakagawa and Fukui (2002), Lau et al. (2006), Miller et al. (2009), and Podar et al. (2020). A recent study has also identified the potential influence of thermal fluctuation on cyanobacterial fitness in hot springs (Hamilton and Havig, 2022). Both the Cyanobacteria and Chloroflexia also include known sulfide-tolerant ecotypes and this must also be viewed as an adaptive advantage in hot springs where sulfide is a common stressor: Sulfide is generally toxic to cyanobacterial primary metabolism because it inhibits cytochrome c oxidase and irreversibly affects the oxygen binding center in photosystem II (Miller and Bebout, 2004). Sulfide-adapted *Oscillatoria* species such as those encountered in our study have been shown *in vitro* to have a sulfide resistant photosystem II that accounts for oxygenic photosynthesis under high sulfide (Martin-Clemente et al., 2022), and other cyanobacteria may facultatively shift from oxygenic to anoxygenic photosynthesis using sulfide as an electron donor and using only photosystem I (Cohen et al., 1975; Hamilton et al., 2018). The distribution of Chloroflexi in hot springs may occur independently from sulfide levels (Hamilton et al., 2019). Hot spring *Chloroflexus* displayed sulfide dependent anoxygenic photosynthesis (Madigan and Brock, 1975), but strains were also capable of sulfide-dependent oxygenic photosynthesis using the 3-hydroxypropionate pathway for C fixation (Kanno et al., 2019). This versatility to perform oxygenic and anoxygenic photosynthesis is likely responsible for the success of these taxa in hot springs. Isotopic incorporation studies have indicated that the relative activity of Cyanobacteria and Chloroflexia is dependent on temperature, with lower temperatures favoring oxygenic photosynthesis and anoxygenic photosynthesis at higher temperatures (Bennett et al., 2020). It is also likely that the two physiologies are linked, with evidence that glyoxylate and lactate are exchanged in mats where both photosynthetic pathways occur (Kim et al., 2015).

## 4. Conclusion

This study has added to the growing global inventory of hot spring microbiomes by characterizing the diversity and interactions in photosynthetic microbial mats and flocs along an environmental stress gradient of temperature and sulfide at Sembawang Hot Spring. The data highlighted the importance of Cyanobacteria at lower temperatures and sulfide levels and Chloroflexia at higher temperatures and sulfide levels in circumneutral pH hot springs. The clear response of diversity to abiotic stressors and the occurrence of discrete groups of taxa sharing biotic interactions suggest that three distinct photosynthetic mat communities arise within the Sembawang Hot Spring habitat. The identification of abundant thermophilic cellulolytic bacteria warrants further research on their ecological role and potential applications. Overall, the findings highlight the need for conservation of hot spring features across environmental gradients in order to maintain the unique biodiversity of their microbiomes. Interesting avenues for future research include measurement of how thermophilic microbial diversity responds to broader poly-extreme environmental gradients across wider biogeographic ranges, and the extent to which the tropical climate and geothermal variation result in temporal variation in communities. This will allow improved estimates of resilience in hot spring microbial communities.

## Data availability statement

The datasets presented in this study can be found in online repositories. The names of the repository/repositories and accession number(s) can be found at: https://www.ncbi.nlm.nih.gov/, PRJNA936476.

## Author contributions

CL and SP conducted the fieldwork. CL and CG performed laboratory experiments. YT performed microscopy. CG and SP analyzed the data and wrote the manuscript. All authors contributed to the article and approved the submitted version.

## Funding

This work was performed in accordance with Singapore National Parks permit NP/RP21-126-1 issued in December 2021. This research was supported by Yale-NUS College seed funding.

## Conflict of interest

The authors declare that the research was conducted in the absence of any commercial or financial relationships that could be construed as a potential conflict of interest.

## Publisher’s note

All claims expressed in this article are solely those of the authors and do not necessarily represent those of their affiliated organizations, or those of the publisher, the editors and the reviewers. Any product that may be evaluated in this article, or claim that may be made by its manufacturer, is not guaranteed or endorsed by the publisher.

## References

[ref1] Albuquerque L. Polónia A. R. M. Barroso C. Froufe H. J. C. Lage O. Lobo-da-Cunha A.. (2018). Raineya orbicola gen. nov., sp. nov. a slightly thermophilic bacterium of the phylum Bacteroidetes and the description of Raineyaceae fam. nov. Int. J. Syst. Evol. Microbiol. 68, 982–989. doi: 10.1099/ijsem.0.002556, PMID: 29458463 PMC5982127

[ref2] Alcorta J. Alarcon-Schumacher T. Salgado O. Diez B. (2020). Taxonomic novelty and distinctive genomic features of hot spring cyanobacteria. Front. Genet. 11:568223. doi: 10.3389/fgene.2020.568223 33250920 PMC7674949

[ref3] Alcorta J. Espinoza S. Viver T. Alcaman-Arias M. E. Trefault N. Rossello-Mora R.. (2018). Temperature modulates *Fischerella thermalis* ecotypes in porcelana hot spring. Syst. Appl. Microbiol. 41, 531–543. doi: 10.1016/j.syapm.2018.05.006, PMID: 30041921

[ref4] Bennett A. C. Murugapiran S. K. Hamilton T. L. (2020). Temperature impacts community structure and function of phototrophic Chloroflexi and cyanobacteria in two alkaline hot springs in Yellowstone National Park. Environ. Microbiol. Rep. 12, 503–513. doi: 10.1111/1758-2229.12863, PMID: 32613733 PMC7540483

[ref5] Boyd E. S. Fecteau K. M. Havig J. R. Shock E. L. Peters J. W. (2012). Modeling the habitat range of phototrophs in yellowstone national park: toward the development of a comprehensive fitness landscape. Front. Microbiol. 3:221. doi: 10.3389/fmicb.2012.00221, PMID: 22719737 PMC3376417

[ref6] Callahan B. J. McMurdie P. J. Rosen M. J. Han A. W. Johnson A. J. A. Holmes S. P. (2016). DADA2: high-resolution sample inference from Illumina amplicon data. Nat. Methods 13, 581–583. doi: 10.1038/nmeth.3869, PMID: 27214047 PMC4927377

[ref7] Caporaso J. G. Lauber C. L. Walters W. A. Berg-Lyons D. Huntley J. Fierer N.. (2012). Ultra-high-throughput microbial community analysis on the Illumina HiSeq and MiSeq platforms. ISME J. 6, 1621–1624. doi: 10.1038/ismej.2012.8, PMID: 22402401 PMC3400413

[ref8] Castenholz R. W. (1976). The effect of sulfide on the bluegreen algae of hot springs. 1. New Zealand and Iceland. J. Phycol. 12, 54–68. doi: 10.1111/j.1529-8817.1976.tb02826.x

[ref9] Castenholz R. W. Utkilen H. C. (1984). Physiology of sulfide tolerance in a thermophilic Oscillatoria. Arch. Microbiol. 138, 299–305. doi: 10.1007/BF00410894

[ref10] Cohen Y. Jørgensen B. B. Padan E. Shilo M. (1975). Sulphide-dependent anoxygenic photosynthesis in the cyanobacterium *Oscillatoria limnetica*. Nature 257, 489–492. doi: 10.1038/257489a0 PMC235807 808537

[ref11] Cox A. Shock E. L. Havig J. R. (2011). The transition to microbial photosynthesis in hot spring ecosystems. Chem. Geol. 280, 344–351. doi: 10.1016/j.chemgeo.2010.11.022

[ref12] De Cáceres M. Legendre P. Moretti M. (2010). Improving indicator species analysis by combining groups of sites. Oikos 119, 1674–1684. doi: 10.1111/j.1600-0706.2010.18334.x

[ref13] Estrella Alcaman M. Fernandez C. Delgado A. Bergman B. Diez B. (2015). The cyanobacterium Mastigocladus fulfills the nitrogen demand of a terrestrial hot spring microbial mat. ISME J. 9, 2290–2303. doi: 10.1038/ismej.2015.63, PMID: 26230049 PMC4579480

[ref14] Fecteau K. M. Boyd E. S. Lindsay M. R. Amenabar M. J. Robinson K. J. Debes R. V. II. (2022). Cyanobacteria and algae meet at the limits of their habitat ranges in moderately acidic hot springs. J. Geophys. Res. Biogeosci. 127:e2021JG006446. doi: 10.1029/2021JG006446

[ref15] Gu Z. (2022). Complex heatmap visualization. iMeta 1:e43. doi: 10.1002/imt2.43 PMC10989952 38868715

[ref16] Gu Z. Eils R. Schlesner M. (2016). Complex heatmaps reveal patterns and correlations in multidimensional genomic data. Bioinformatics 32, 2847–2849. doi: 10.1093/bioinformatics/btw313, PMID: 27207943

[ref17] Hallenbeck P. C. Grogger M. Mraz M. Veverka D. (2016). Draft genome sequence of a thermophilic cyanobacterium from the family <i>Oscillatoriales</i> (strain MTP1) from the Chalk River, Colorado. Genome Announc 4:e01571-15. doi: 10.1128/genomeA.01571-15, PMID: 26893415 PMC4759062

[ref18] Hamilton T. L. Bennett A. C. Murugapiran S. K. Havig J. R. (2019). Anoxygenic phototrophs span geochemical gradients and diverse morphologies in terrestrial geothermal springs. mSystems 4:e00498-19. doi: 10.1128/mSystems.00498-19 31690593 PMC6832021

[ref19] Hamilton T. L. Havig J. (2022). Meet me in the middle: median temperatures impact cyanobacteria and photoautotrophy in eruptive yellowstone hot springs. mSystems 7:e0145021. doi: 10.1128/msystems.01450-21, PMID: 35089080 PMC8725584

[ref20] Hamilton T. L. Klatt J. M. de Beer D. Macalady J. L. (2018). Cyanobacterial photosynthesis under sulfidic conditions: insights from the isolate Leptolyngbya sp. strain hensonii. ISME J. 12, 568–584. doi: 10.1038/ismej.2017.193, PMID: 29328062 PMC5776472

[ref21] Hanada S. (2014). “The phylum chloroflexi, the family chloroflexaceae, and the related phototrophic families oscillochloridaceae and roseiflexaceae” in The prokaryotes. eds. Rosenberg E. DeLong E. F. Lory S. Stackebrandt E. Thompson F. (Berlin Heidelberg: Springer), 515–532.

[ref22] Jing H. Aitchison J. C. Lacap D. C. Peerapornpisal Y. Sompong U. Pointing S. B. (2005). Community phylogenetic analysis of moderately thermophilic cyanobacterial mats from China, the Philippines and Thailand. Extremophiles 9, 325–332. doi: 10.1007/s00792-005-0456-1, PMID: 15970994

[ref23] Jing H. Lacap D. C. Lau C. Y. Pointing S. B. (2006). Community phylogenetic diversity of cyanobacterial mats associated with geothermal springs along a tropical intertidal gradient. Extremophiles 10, 159–163. doi: 10.1007/s00792-005-0477-9, PMID: 16143880

[ref24] Kanno N. Haruta S. Hanada S. (2019). Sulfide-dependent photoautotrophy in the filamentous anoxygenic phototrophic bacterium, *Chloroflexus aggregans*. Microbes Environ 34, 304–309. doi: 10.1264/jsme2.ME19008, PMID: 31391357 PMC6759344

[ref25] Kawai S. Kamiya N. Matsuura K. Haruta S. (2019). Symbiotic growth of a thermophilic sulfide-oxidizing photoautotroph and an elemental sulfur-disproportionating chemolithoautotroph and cooperative dissimilatory oxidation of sulfide to sulfate. Front. Microbiol. 10:1150. doi: 10.3389/fmicb.2019.01150 31178849 PMC6543001

[ref26] Kees E. D. Murugapiran S. K. Bennett A. C. Hamilton T. L. (2022). Distribution and genomic variation of thermophilic cyanobacteria in diverse microbial mats at the upper temperature limits of photosynthesis. mSystems 7:e0031722. doi: 10.1128/msystems.00317-22, PMID: 35980085 PMC9600594

[ref27] Keshari N. Zhao Y. Das S. K. Zhu T. Lu X. (2022). Cyanobacterial community structure and isolates from representative hot springs of Yunnan Province, China using an integrative approach. Front. Microbiol. 13:872598. doi: 10.3389/fmicb.2022.872598, PMID: 35547135 PMC9083006

[ref28] Kim Y. M. Nowack S. Olsen M. T. Becraft E. D. Wood J. M. Thiel V.. (2015). Diel metabolomics analysis of a hot spring chlorophototrophic microbial mat leads to new hypotheses of community member metabolisms. Front. Microbiol. 6:209. doi: 10.3389/fmicb.2015.00209, PMID: 25941514 PMC4400912

[ref29] Klatt C. G. Wood J. M. Rusch D. B. Bateson M. M. Hamamura N. Heidelberg J. F.. (2011). Community ecology of hot spring cyanobacterial mats: predominant populations and their functional potential. ISME J. 5, 1262–1278. doi: 10.1038/ismej.2011.73, PMID: 21697961 PMC3146275

[ref30] Lacap D. C. Barraquio W. Pointing S. B. (2007). Thermophilic microbial mats in a tropical geothermal location display pronounced seasonal changes but appear resilient to stochastic disturbance. Environ. Microbiol. 9, 3065–3076. doi: 10.1111/j.1462-2920.2007.01417.x, PMID: 17991034

[ref31] Lau M. C. Aitchison J. C. Pointing S. B. (2009). Bacterial community composition in thermophilic microbial mats from five hot springs in Central Tibet. Extremophiles 13, 139–149. doi: 10.1007/s00792-008-0205-3, PMID: 19023516

[ref32] Lau C. Y. Jing H. Aitchison J. C. Pointing S. B. (2006). Highly diverse community structure in a remote central Tibetan geothermal spring does not display monotonic variation to thermal stress. FEMS Microbiol. Ecol. 57, 80–91. doi: 10.1111/j.1574-6941.2006.00104.x, PMID: 16819952

[ref33] Li J. Gao R. Chen Y. Xue D. Han J. Wang J.. (2020). Isolation and identification of *Microvirga thermotolerans* HR1, a novel thermo-tolerant bacterium, and comparative genomics among microvirga species. Microorganisms 8:101. doi: 10.3390/microorganisms8010101, PMID: 31936875 PMC7022394

[ref34] Lino T. Mori K. Uchino Y. Nakagawa T. Harayama S. Suzuki K. I. (2010). *Ignavibacterium album* gen. nov., sp. nov., a moderately thermophilic anaerobic bacterium isolated from microbial mats at a terrestrial hot spring and proposal of Ignavibacteria classis nov., for a novel lineage at the periphery of green sulfur bacteria. Int. J. Syst. Evol. Microbiol. 60, 1376–1382. doi: 10.1099/ijs.0.012484-0 19671715

[ref35] Ma Z. Gao K. (2009). Photoregulation of morphological structure and its physiological relevance in the cyanobacterium Arthrospira (Spirulina) platensis. Planta 230, 329–337. doi: 10.1007/s00425-009-0947-x, PMID: 19466449

[ref36] Madigan M. T. Brock T. D. (1975). Photosynthetic sulfide oxidation by *Chloroflexus aurantiacus*, a filamentous, photosynthetic, gliding bacterium. J. Bacteriol. 122, 782–784. doi: 10.1128/jb.122.2.782-784.1975, PMID: 1092670 PMC246117

[ref37] Martin-Clemente E. Melero-Jimenez I. J. Banares-Espana E. Flores-Moya A. Garcia-Sanchez M. J. (2022). Photosynthetic performance in cyanobacteria with increased sulphide tolerance: an analysis comparing wild-type and experimentally derived strains. Photosynth. Res. 151, 251–263. doi: 10.1007/s11120-021-00882-8, PMID: 34807429 PMC8940870

[ref38] Martinez J. N. Nishihara A. Lichtenberg M. Trampe E. Kawai S. Tank M.. (2019). Vertical distribution and diversity of phototrophic bacteria within a hot spring microbial mat (Nakabusa Hot Springs, Japan). Microbes Environ. 34, 374–387. doi: 10.1264/jsme2.ME19047, PMID: 31685759 PMC6934398

[ref39] Miller S. R. Bebout B. M. (2004). Variation in sulfide tolerance of photosystem II in phylogenetically diverse cyanobacteria from sulfidic habitats. Appl. Environ. Microbiol. 70, 736–744. doi: 10.1128/aem.70.2.736-744.2004, PMID: 14766549 PMC348820

[ref40] Miller S. R. Strong A. L. Jones K. L. Ungerer M. C. (2009). Bar-coded pyrosequencing reveals shared bacterial community properties along the temperature gradients of two alkaline hot springs in Yellowstone National Park. Appl. Environ. Microbiol. 75, 4565–4572. doi: 10.1128/AEM.02792-08, PMID: 19429553 PMC2704827

[ref41] Nakagawa T. Fukui M. (2002). Phylogenetic characterization of microbial mats and streamers from a Japanese alkaline hot spring with a thermal gradient. J. Gen. Appl. Microbiol. 48, 211–222. doi: 10.2323/jgam.48.211, PMID: 12469320

[ref42] Oksanen J. Blanchet F. G. Friendly M. Kindt R. Legendre P. McGlinn D.., (2020). Vegan: community ecology package. Available at: https://CRAN.R-project.org/package=vegan

[ref43] Papke R. T. Ramsing N. B. Bateson M. M. Ward D. M. (2003). Geographical isolation in hot spring cyanobacteria. Environ. Microbiol. 5, 650–659. doi: 10.1046/j.1462-2920.2003.00460.x, PMID: 12871232

[ref44] Peschel S. Muller C. L. von Mutius E. Boulesteix A. L. Depner M. (2021). NetCoMi: network construction and comparison for microbiome data in R. Brief. Bioinform. 22:bbaa290. doi: 10.1093/bib/bbaa290 33264391 PMC8293835

[ref45] Podar P. T. Yang Z. Bjornsdottir S. H. Podar M. (2020). Comparative analysis of microbial diversity across temperature gradients in hot springs from Yellowstone and Iceland. Front. Microbiol. 11:1625. doi: 10.3389/fmicb.2020.01625, PMID: 32760379 PMC7372906

[ref46] Podosokorskaya O. A. Merkel A. Y. Kolganova T. V. Chernyh N. A. Miroshnichenko M. L. Bonch-Osmolovskaya E. A.. (2011). *Fervidobacterium riparium* sp. nov., a thermophilic anaerobic cellulolytic bacterium isolated from a hot spring. Int. J. Syst. Evol. Microbiol. 61, 2697–2701. doi: 10.1099/ijs.0.026070-0 21169457

[ref47] Power J. F. Carere C. R. Lee C. K. Wakerley G. L. J. Evans D. W. Button M.. (2018). Microbial biogeography of 925 geothermal springs in New Zealand. Nat. Commun. 9:2876. doi: 10.1038/s41467-018-05020-y, PMID: 30038374 PMC6056493

[ref48] Purcell D. Sompong U. Yim L. C. Barraclough T. G. Peerapornpisal Y. Pointing S. B. (2007). The effects of temperature, pH and sulphide on the community structure of hyperthermophilic streamers in hot springs of northern Thailand. FEMS Microbiol. Ecol. 60, 456–466. doi: 10.1111/j.1574-6941.2007.00302.x, PMID: 17386034

[ref49] Quast C. Pruesse E. Yilmaz P. Gerken J. Schweer T. Yarza P.. (2013). The SILVA ribosomal RNA gene database project: improved data processing and web-based tools. Nucleic Acids Res. 41, D590–D596. doi: 10.1093/nar/gks1219, PMID: 23193283 PMC3531112

[ref50] R Core Team. (2021). A language and environment for statistical computing. R foundation for statistical computing. Vienna.

[ref51] Rakitin A. L. Naumoff D. G. Beletsky A. V. Kulichevskaya I. S. Mardanov A. V. Ravin N. V.. (2021). Complete genome sequence of the cellulolytic planctomycete Telmatocola sphagniphila SP2(T) and characterization of the first cellulolytic enzyme from planctomycetes. Syst. Appl. Microbiol. 44:126276. doi: 10.1016/j.syapm.2021.126276, PMID: 34735803

[ref52] Rozanov A. S. Bryanskaya A. V. Ivanisenko T. V. Malup T. K. Peltek S. E. (2017). Biodiversity of the microbial mat of the Garga hot spring. BMC Evol. Biol. 17:254. doi: 10.1186/s12862-017-1106-9, PMID: 29297382 PMC5751763

[ref53] Sato N. Katsumata Y. Sato K. Tajima N. (2014). Cellular dynamics drives the emergence of supracellular structure in the cyanobacterium, Phormidium sp. KS. Life 4, 819–836. doi: 10.3390/life4040819, PMID: 25460162 PMC4284469

[ref54] Schuler C. G. Havig J. R. Hamilton T. L. (2017). Hot spring microbial community composition, morphology, and carbon fixation: implications for interpreting the ancient rock record. Front. Earth Sci. 5:97. doi: 10.3389/feart.2017.00097

[ref55] Shu W.-S. Huang L.-N. (2022). Microbial diversity in extreme environments. Nat. Rev. Microbiol. 20, 219–235. doi: 10.1038/s41579-021-00648-y 34754082

[ref56] Sompong U. Hawkins P. R. Besley C. Peerapornpisal Y. (2005). The distribution of cyanobacteria across physical and chemical gradients in hot springs in northern Thailand. FEMS Microbiol. Ecol. 52, 365–376. doi: 10.1016/j.femsec.2004.12.007, PMID: 16329921

[ref57] Steunou A.-S. Bhaya D. Bateson M. M. Melendrez M. C. Ward D. M. Brecht E.. (2006). In situ analysis of nitrogen fixation and metabolic switching in unicellular thermophilic cyanobacteria inhabiting hot spring microbial mats. Proc. Natl. Acad. Sci. 103, 2398–2403. doi: 10.1073/pnas.0507513103, PMID: 16467157 PMC1413695

[ref58] Subudhi E. Sahoo R. K. Gaur M. Singh A. Das A. (2018). Shift in cyanobacteria community diversity in hot springs of India. Geomicrobiol J. 35, 141–147. doi: 10.1080/01490451.2017.1338799

[ref59] Tang J. Jiang D. Luo Y. Liang Y. Li L. Shah M. M. R.. (2018). Potential new genera of cyanobacterial strains isolated from thermal springs of western Sichuan, China. Algal Res. 31, 14–20. doi: 10.1016/j.algal.2018.01.008

[ref60] Thiel V. Wood J. M. Olsen M. T. Tank M. Klatt C. G. Ward D. M.. (2016). The dark side of the mushroom spring microbial mat: life in the shadow of chlorophototrophs. I. Microbial diversity based on 16S rRNA gene amplicons and metagenomic sequencing. Front. Microbiol. 7:919. doi: 10.3389/fmicb.2016.00919, PMID: 27379049 PMC4911352

[ref61] Thompson L. R. Sanders J. G. McDonald D. Amir A. Ladau J. Locey K. J.. (2017). A communal catalogue reveals Earth’s multiscale microbial diversity. Nature 551, 457–463. doi: 10.1038/nature24621, PMID: 29088705 PMC6192678

[ref62] Upin H. E. Newell D. L. Colman D. R. Boyd E. S. (2023). Tectonic settings influence the geochemical and microbial diversity of Peru hot springs. Commun. Earth Environ. 4:112. doi: 10.1038/s43247-023-00787-5 PMC11041657 38665187

[ref63] Wang R. Lin J. Q. Liu X. M. Pang X. Zhang C. J. Yang C. L.. (2018). Sulfur oxidation in the acidophilic autotrophic Acidithiobacillus spp. Front. Microbiol. 9:3290. doi: 10.3389/fmicb.2018.03290, PMID: 30687275 PMC6335251

[ref64] Wurch L. Giannone R. J. Belisle B. S. Swift C. Utturkar S. Hettich R. L.. (2016). Genomics-informed isolation and characterization of a symbiotic Nanoarchaeota system from a terrestrial geothermal environment. Nat. Commun. 7:12115. doi: 10.1038/ncomms12115, PMID: 27378076 PMC4935971

[ref65] Zhao J. Chen C. N. Cai J. G. (2001). A hydrogeological study of the Sembawang Hot Spring in Singapore. Bull. Eng. Geol. Environ. 61, 59–71. doi: 10.1007/s10064-001-0143-0

[ref66] Zhao C. Li Z. Li T. Zhang Y. Bryant D. A. Zhao J. (2015). High-yield production of extracellular type-I cellulose by the cyanobacterium Synechococcus sp. PCC 7002. Cell Discov 1:15004. doi: 10.1038/celldisc.2015.4, PMID: 27462405 PMC4851311

