# Supplementary material for: Community structure of thermophilic photosynthetic microbial mats and flocs at Sembawang Hot Spring, Singapore
Source: Front Microbiol. 2023 Jun 16;14:1189468. doi: 10.3389/fmicb.2023.1189468 (PMC10313338; doi:10.3389/fmicb.2023.1189468)

Supplementary Material

Community structure of thermophilic photosynthetic microbial mats and flocs at Sembawang Hot Spring, Singapore

Christaline George[1], Chloe Xue Qi Lim[1], Yan Tong[2], Stephen Brian Pointing[1,2*]
[1] Yale-NUS College, National University of Singapore, Singapore
[2] Department of Biological Sciences, National University of Singapore, Singapore
* Correspondence: Stephen Pointing, yncpsb@nus.edu.sg

Table S1. Aqueous geochemical variables at Sembawang Hot Spring. N/D denotes not below detection limits.

Location	Lower channel	Mid channel	Upper channel	Source
Growth form	Mat	Mat	Mat	Floc
Sample codes	A1-A10, ABR1-ABR3	B1-B10, BBR1-BBR3	C1-C10, CBR1-CBR3	D1-D10, DBR1-DBR3
Temperature (°C)	45.7	51.0	55.3	61.4
Sulfide (mg/L)	0.0	0.3	0.5	1.0
pH	6.8	6.8	6.8	6.8
Alkalinity (mg/L)	40	40	40	40
Carbonate (mg/L)	40	40	40	80
Conductivity (µS)	1,920	1,900	1,920	1,905
Iron (mg/L)	N/D	N/D	N/D	N/D
Nitrate (mg/L)	N/D	N/D	N/D	N/D
Nitrite (mg/L)	N/D	N/D	N/D	N/D
Phosphate (ng/L)	100	100	100	100

Table S2. Taxonomic assignment of ASVs with $\geq$1% relative abundance in at least one sample using the SILVA 138 database, and additional query where necessary using NCBI BLASTn.

**ASV number**	**Class**	**ASV identity**
ASV0	Cyanobacteriia	MTP1
ASV1	Chloroflexia	*Chloroflexus* sp.
ASV2	Verrucomicrobiae	Methylacidiphilaceae
ASV3	Chloroflexia	*Roseiflexus* sp.
ASV4	Cyanobacteriia	*Thermosynechococcus elongatus*
ASV5	Bacteroidia	*Raineya orbicola*
ASV6	Ignavibacteria	*Ignavibacterium album*
ASV7	Gammaproteobacteria	Hydrogenophilaceae
ASV8	Bacteroidia	Microsillaceae
ASV9	Gammaproteobacteria	*Tepidomonas* sp.
ASV10	Chloroflexia	*Candidatus Chloroploca* sp.
ASV108	Synergistia	*Aminiphilus* sp.
ASV11	Chloroflexia	*Chloroflexus aurantiacus*
ASV114	Nitrosphaeria	*Candidatus Nitrosotenuis aquarius*
ASV12	Thermodeslufovibrionia	*Thermodesulfovibrio* sp.
ASV13	Planctomyectes	*Telmatocola* sp.
ASV138	Bacteroidia	Blvii_watsewater-sludge_group sp.
ASV14	Gammaproteobacteria	*Methylothermus* sp.
ASV15	Alphaproteobacteria	Alphaproteobacteria
ASV155	Thermotogae	*Fervidobacterium riparium*
ASV16	Myxococcia	Myxococcaceae

ASV17	Bacteroidia	Saprospiraceae
ASV18	Chloroflexia	Chloroflexaceae
ASV19	Bacteroidia	Microsillaceae
ASV20	Anaerolineae	Anaerolineaceae
ASV21	Chloroflexia	*Roseiflexus* sp.
ASV22	Anaerolineae	OLB13 sp.
ASV23	Leptospirae	*Turneriella* sp.
ASV24	Cyanobacteriia	*Fischerella* sp. PCC9339
ASV25	Bacteroidia	Bacteroidia
ASV26	Gammaproteobacteria	DSSD61 sp.
ASV27	Bacteroidia	Bacteroidales
ASV28	Bacteria	Unidentified sp.
ASV29	Armatimonadota	Hot springs ASV
ASV30	Alphaproteobacteria	*Microvirga* sp.
ASV31	Bacteroidia	*Ignavibacterium album*
ASV32	Chloroflexia	*Chloroflexus* sp.
ASV33	Alphaproteobacteria	Alphaproteobacteria
ASV34	Chloroflexia	*Chloroflexus* sp.
ASV35	Acidobacteriae	*Paludibaculum* sp.
ASV36	Gammaproteobacteria	Comamonadaceae
ASV37	Bacteroidia	*Thermoflexibacter ruber*
ASV38	Gammaproteobacteria	Sutterelaceae
ASV39	Anaerolineae	SJA-15 sp.
ASV40	Gammaproteobacteria	Gammaproteobacteria
ASV41	Aquificae	*Venevibrio* sp.
ASV42	Gammaproteobacteria	Gammaproteobacteria
ASV43	Deinococci	*Meiothermus hypogaeus*
ASV44	Spirochaetota	Spirochaetota
ASV45	Cyanobacteriia	*Geitlerinema* PCC-8501
ASV46	Polyangia	mle1-27
ASV47	Ignavibacteria	*Ignavibacterium album*
ASV48	Anaerolineae	RBG-13-54-9 sp.
ASV50	Alphaproteobacteria	*Sandaracinobacter* sp.
ASV51	Anaerolineae	A4b sp.
ASV52	Alphaproteobacteria	Alphaproteobacteria
ASV53	Planctomycetes	*Gemmata* sp.
ASV54	Gemmatimonadetes	Gemmatimonadaceae
ASV55	Bacteria	Unidentified sp.
ASV56	Bacteroidia	Bacteroidia
ASV57	Bacteroidia	Cytophagales
ASV58	Thermodeslufovibrionia	*Thermodesulfovibrio* sp.
ASV59	Phycisphaerae	WD2101 sp.
ASV61	Kryptonia	BSV26 sp.
ASV62	Alphaproteobacteria	Alphaprotoebacteria
ASV63	Acidobacteriae	Acidobacteriae
ASV64	Gammaproteobacteria	Hydrogenophilaceae
ASV65	Alphaproteobacteria	*Candidatus Alysosphaera* sp.
ASV66	Chloroflexia	*Roseiflexus* sp.
ASV68	Nanoarchaeia	*Woesarchaeales* sp.

ASV71	Anaerolineae	A4b sp.
ASV72	Gammaproteobacteria	Hydrogenophilaceae
ASV73	Planctomycetes	Gemmataceae
ASV75	Gemmatimonadetes	Gemmatimonadetes
ASV77	Deinococci	*Meiothermus hypogaeus*
ASV81	Alphaproteobacteria	*Defluvicoccus* sp.
ASV82	Anaerolineae	RGB-13-54-9 sp.
ASV84	Kapabacteria	Kapabacteriales
ASV85	Bacilli	*Streptococcus* sp.
ASV87	Cyanobacteriia	*Leptolyngbya* ANT.L52.2 sp.
ASV88	Bacteroidia	*Petrimonas* sp.
ASV89	Spirochaetia	Spirocaetaceae
ASV90	Bacteroidia	Bacteroidia
ASV93	Verrucomicrobiae	Methylacidiphilaceae
ASV97	Armatimonadota	Armatimonadota
ASV98	Cyanobacteriia	*Leptolyngbya* sp.

Fig. S1. Distribution of ASVs with ≥1% relative abundance at Sembawang Hot Spring.

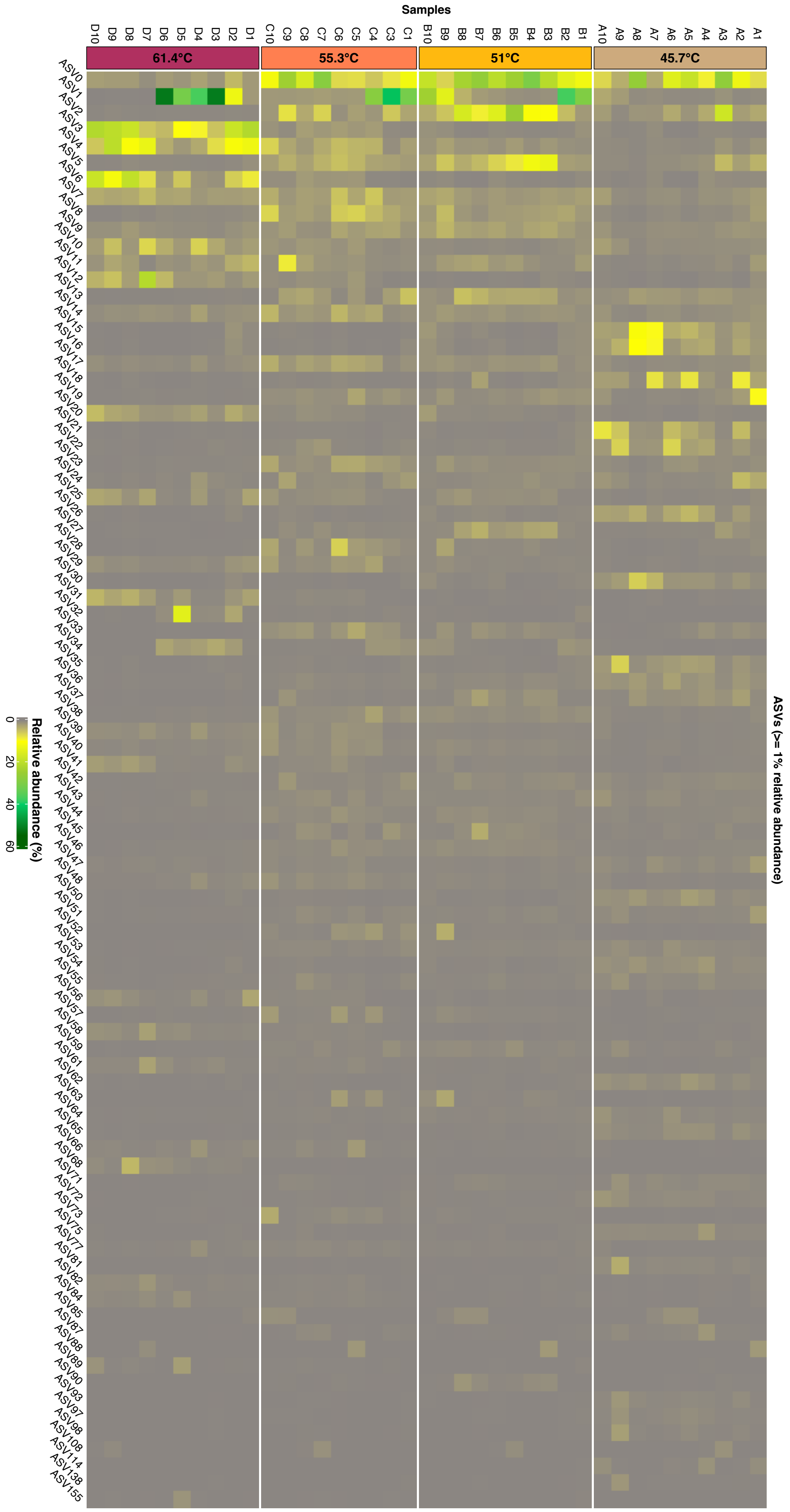

Supplement: Supplementary file 1 [file Data_Sheet_1.PDF]
